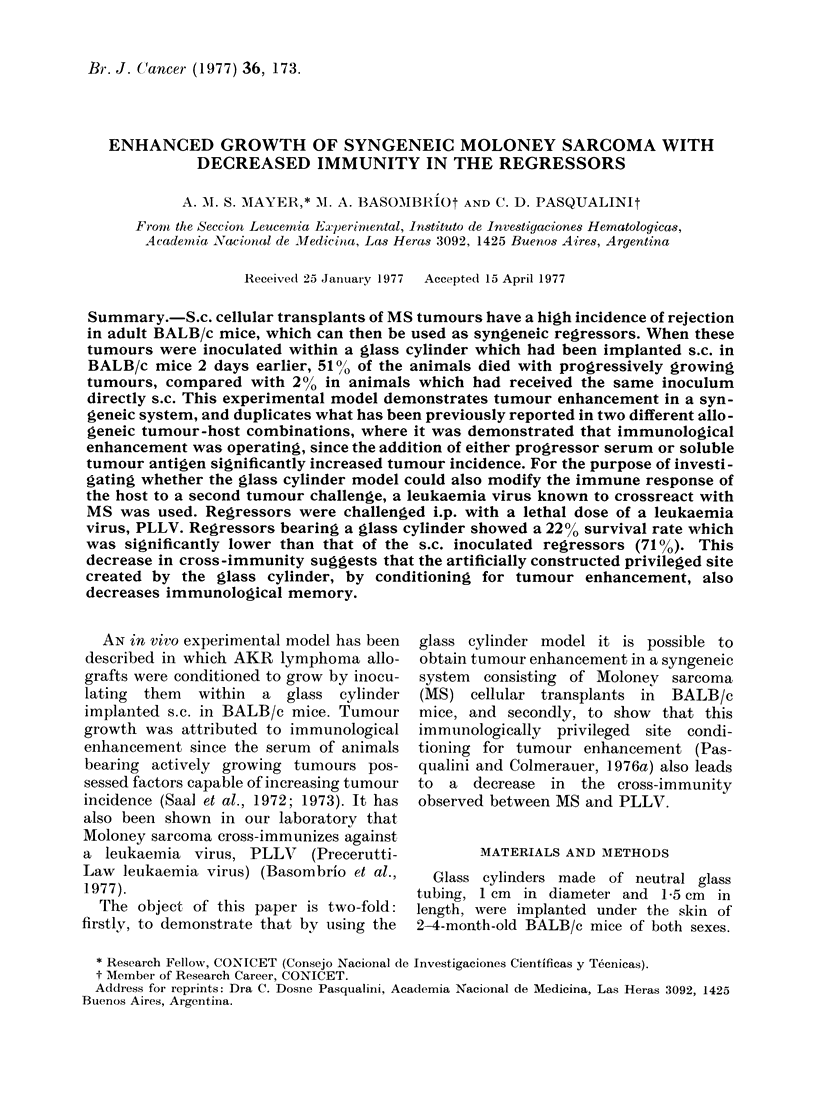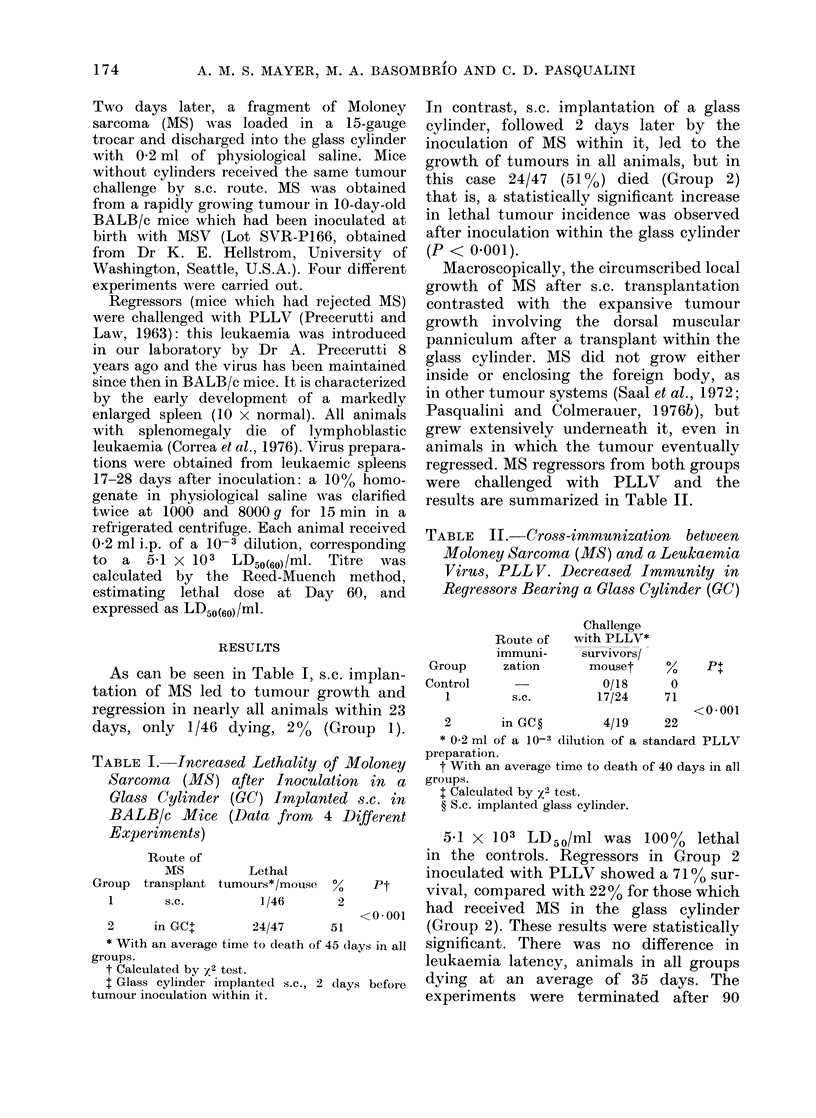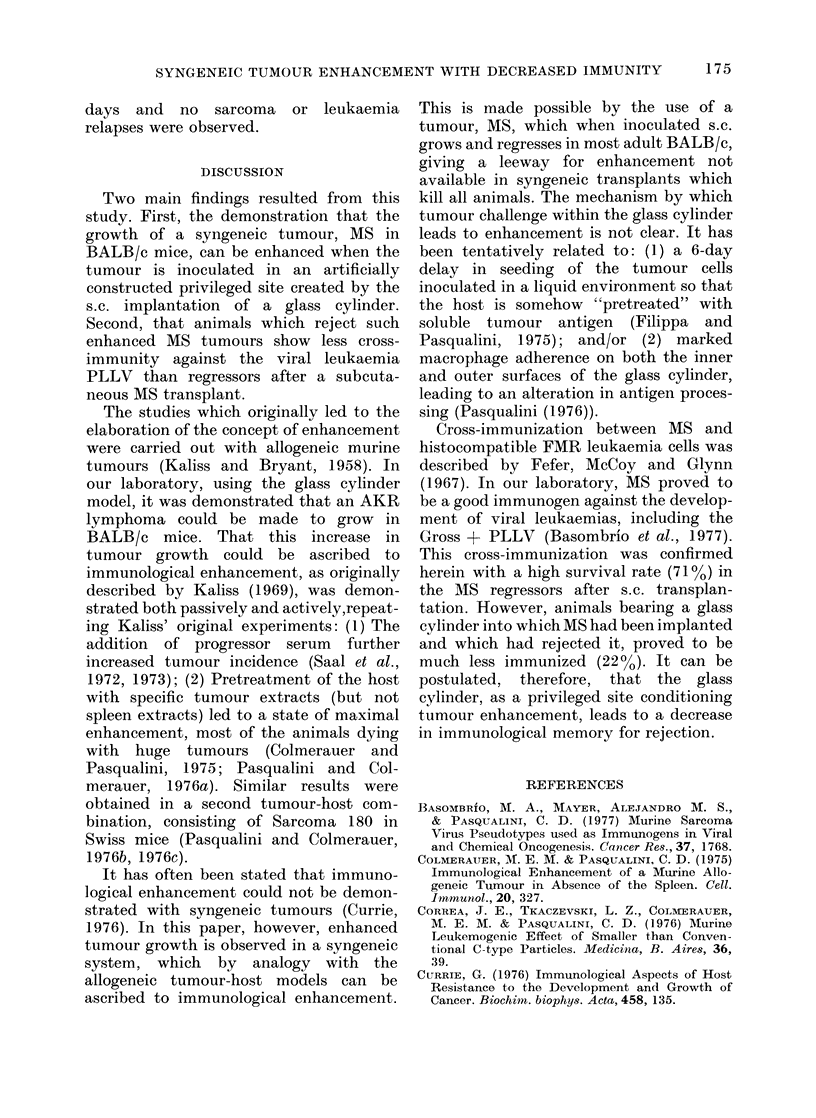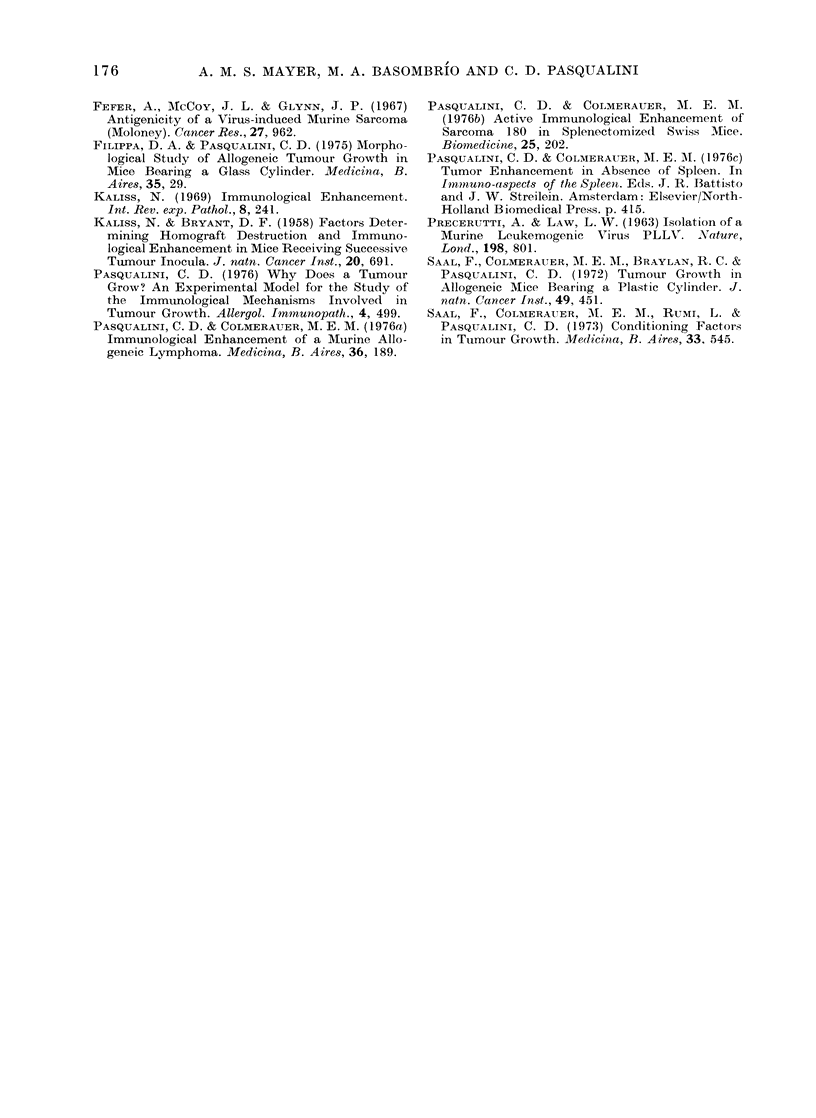# Enhanced Growth of Syngeneic Moloney Sarcoma with Decreased Immunity in the Regressors

**DOI:** 10.1038/bjc.1977.174

**Published:** 1977-08

**Authors:** A. M. S. Mayer, M. A. Basombrío, C. D. Pasqualini

## Abstract

S.c. cellular transplants of MS tumours have a high incidence of rejection in adult BALB/c mice, which can then be used as syngeneic regressors. When these tumours were inoculated within a glass cylinder which had been implanted s.c. in BALB/c mice 2 days earlier, 51% of the animals died with progressively growing tumours, compared with 2% in animals which had received the same inoculum directly s.c. This experimental model demonstrates tumour enhancement in a syngeneic system, and duplicates what has been previously reported in two different allogeneic tumour-host combinations, where it was demonstrated that immunological enhancement was operating, since the addition of either progressor serum or soluble tumour antigen significantly increased tumour incidence. For the purpose of investigating whether the glass cylinder model could also modify the immune response of the host to a second tumour challenge, a leukaemia virus known to crossreact with MS was used. Regressors were challenged i.p. with a lethal dose of a leukaemia virus, PLLV. Regressors bearing a glass cylinder showed a 22% survival rate which was significantly lower than that of the s.c. inoculated regressors (71%). This decrease in cross-immunity suggests that the artificially constructed privileged site created by the glass cylinder, by conditioning for tumour enhancement, also decreases immunological memory.


*Br. J. Cancer* (1977) **36**, 173.

### ENHANCED GROWTH OF SYNGENEIC MOLONEY SARCOMA WITH DECREASED IMMUNITY IN THE REGRESSORS

A. M. S. MAYER,* M. A. BASOMBRÍO† AND C. D. PASQUALINI†

*From the Seccion Leucemia Experimental, Instituto de Investigaciones Hematologicas, Academia Nacional de Medicina, Las Heras* 3092, 1425 *Buenos Aires, Argentina*

Received 25 January 1977   Accepted 15 April 1977

**Summary.—S.c. cellular transplants of MS tumours have a high incidence of rejection in adult BALB/c mice, which can then be used as syngeneic regressors. When these tumours were inoculated within a glass cylinder which had been implanted s.c. in BALB/c mice 2 days earlier, 51% of the animals died with progressively growing tumours, compared with 2% in animals which had received the same inoculum directly s.c. This experimental model demonstrates tumour enhancement in a syngeneic system, and duplicates what has been previously reported in two different allogeneic tumour-host combinations, where it was demonstrated that immunological enhancement was operating, since the addition of either progressor serum or soluble tumour antigen significantly increased tumour incidence. For the purpose of investigating whether the glass cylinder model could also modify the immune response of the host to a second tumour challenge, a leukaemia virus known to crossreact with MS was used. Regressors were challenged i.p. with a lethal dose of a leukaemia virus, PLLV. Regressors bearing a glass cylinder showed a 22% survival rate which was significantly lower than that of the s.c. inoculated regressors (71%). This decrease in cross-immunity suggests that the artificially constructed privileged site created by the glass cylinder, by conditioning for tumour enhancement, also decreases immunological memory.**

AN *in vivo* experimental model has been described in which AKR lymphoma allografts were conditioned to grow by inoculating them within a glass cylinder implanted s.c. in BALB/c mice. Tumour growth was attributed to immunological enhancement since the serum of animals bearing actively growing tumours possessed factors capable of increasing tumour incidence (Saal *et al.*, 1972; 1973). It has also been shown in our laboratory that Moloney sarcoma cross-immunizes against a leukaemia virus, PLLV (Precerutti-Law leukaemia virus) (Basombrío *et al.*, 1977).

The object of this paper is two-fold: firstly, to demonstrate that by using the glass cylinder model it is possible to obtain tumour enhancement in a syngeneic system consisting of Moloney sarcoma (MS) cellular transplants in BALB/c mice, and secondly, to show that this immunologically privileged site conditioning for tumour enhancement (Pasqualini and Colmerauer, 1976*a*) also leads to a decrease in the cross-immunity observed between MS and PLLV.

### MATERIALS AND METHODS

Glass cylinders made of neutral glass tubing, 1 cm in diameter and 1·5 cm in length, were implanted under the skin of 2–4-month-old BALB/c mice of both sexes.

* Research Fellow, CONICET (Consejo Nacional de Investigaciones Científicas y Técnicas).
† Member of Research Career, CONICET.
Address for reprints: Dra C. Dosne Pasqualini, Academia Nacional de Medicina, Las Heras 3092, 1425 Buenos Aires, Argentina.

Two days later, a fragment of Moloney sarcoma (MS) was loaded in a 15-gauge trocar and discharged into the glass cylinder with 0·2 ml of physiological saline. Mice without cylinders received the same tumour challenge by s.c. route. MS was obtained from a rapidly growing tumour in 10-day-old BALB/c mice which had been inoculated at birth with MSV (Lot SVR-P166, obtained from Dr K. E. Hellstrom, University of Washington, Seattle, U.S.A.). Four different experiments were carried out.

Regressors (mice which had rejected MS) were challenged with PLLV (Precerutti and Law, 1963): this leukaemia was introduced in our laboratory by Dr A. Precerutti 8 years ago and the virus has been maintained since then in BALB/c mice. It is characterized by the early development of a markedly enlarged spleen (10 × normal). All animals with splenomegaly die of lymphoblastic leukaemia (Correa *et al.*, 1976). Virus preparations were obtained from leukaemic spleens 17–28 days after inoculation: a 10% homogenate in physiological saline was clarified twice at 1000 and 8000 *g* for 15 min in a refrigerated centrifuge. Each animal received 0·2 ml i.p. of a $10^{-3}$ dilution, corresponding to a $5{\cdot}1 \times 10^3$ $LD_{50(60)}$/ml. Titre was calculated by the Reed-Muench method, estimating lethal dose at Day 60, and expressed as $LD_{50(60)}$/ml.

### RESULTS

As can be seen in Table I, s.c. implantation of MS led to tumour growth and regression in nearly all animals within 23 days, only 1/46 dying, 2% (Group 1).

TABLE I.—*Increased Lethality of Moloney Sarcoma (MS) after Inoculation in a Glass Cylinder (GC) Implanted s.c. in BALB/c Mice (Data from 4 Different Experiments)*

| Group | Route of MS transplant | Lethal tumours*/mouse | % | P† |
|---|---|---|---|---|
| 1 | s.c. | 1/46 | 2 | |
| | | | | <0·001 |
| 2 | in GC‡ | 24/47 | 51 | |

* With an average time to death of 45 days in all groups.

† Calculated by $\chi^2$ test.

‡ Glass cylinder implanted s.c., 2 days before tumour inoculation within it.

In contrast, s.c. implantation of a glass cylinder, followed 2 days later by the inoculation of MS within it, led to the growth of tumours in all animals, but in this case 24/47 (51%) died (Group 2) that is, a statistically significant increase in lethal tumour incidence was observed after inoculation within the glass cylinder ($P < 0{\cdot}001$).

Macroscopically, the circumscribed local growth of MS after s.c. transplantation contrasted with the expansive tumour growth involving the dorsal muscular panniculum after a transplant within the glass cylinder. MS did not grow either inside or enclosing the foreign body, as in other tumour systems (Saal *et al.*, 1972; Pasqualini and Colmerauer, 1976*b*), but grew extensively underneath it, even in animals in which the tumour eventually regressed. MS regressors from both groups were challenged with PLLV and the results are summarized in Table II.

TABLE II.—*Cross-immunization between Moloney Sarcoma (MS) and a Leukaemia Virus, PLLV. Decreased Immunity in Regressors Bearing a Glass Cylinder (GC)*

| Group | Route of immunization | Challenge with PLLV* survivors/mouse† | % | P‡ |
|---|---|---|---|---|
| Control | — | 0/18 | 0 | |
| 1 | s.c. | 17/24 | 71 | |
| | | | | <0·001 |
| 2 | in GC§ | 4/19 | 22 | |

* 0·2 ml of a $10^{-3}$ dilution of a standard PLLV preparation.
† With an average time to death of 40 days in all groups.
‡ Calculated by $\chi^2$ test.
§ S.c. implanted glass cylinder.

$5{\cdot}1 \times 10^3$ $LD_{50}$/ml was 100% lethal in the controls. Regressors in Group 2 inoculated with PLLV showed a 71% survival, compared with 22% for those which had received MS in the glass cylinder (Group 2). These results were statistically significant. There was no difference in leukaemia latency, animals in all groups dying at an average of 35 days. The experiments were terminated after 90

days and no sarcoma or leukaemia relapses were observed.

### DISCUSSION

Two main findings resulted from this study. First, the demonstration that the growth of a syngeneic tumour, MS in BALB/c mice, can be enhanced when the tumour is inoculated in an artificially constructed privileged site created by the s.c. implantation of a glass cylinder. Second, that animals which reject such enhanced MS tumours show less cross-immunity against the viral leukaemia PLLV than regressors after a subcutaneous MS transplant.

The studies which originally led to the elaboration of the concept of enhancement were carried out with allogeneic murine tumours (Kaliss and Bryant, 1958). In our laboratory, using the glass cylinder model, it was demonstrated that an AKR lymphoma could be made to grow in BALB/c mice. That this increase in tumour growth could be ascribed to immunological enhancement, as originally described by Kaliss (1969), was demonstrated both passively and actively, repeating Kaliss' original experiments: (1) The addition of progressor serum further increased tumour incidence (Saal *et al.*, 1972, 1973); (2) Pretreatment of the host with specific tumour extracts (but not spleen extracts) led to a state of maximal enhancement, most of the animals dying with huge tumours (Colmerauer and Pasqualini, 1975; Pasqualini and Colmerauer, 1976*a*). Similar results were obtained in a second tumour-host combination, consisting of Sarcoma 180 in Swiss mice (Pasqualini and Colmerauer, 1976*b*, 1976*c*).

It has often been stated that immunological enhancement could not be demonstrated with syngeneic tumours (Currie, 1976). In this paper, however, enhanced tumour growth is observed in a syngeneic system, which by analogy with the allogeneic tumour-host models can be ascribed to immunological enhancement. This is made possible by the use of a tumour, MS, which when inoculated s.c. grows and regresses in most adult BALB/c, giving a leeway for enhancement not available in syngeneic transplants which kill all animals. The mechanism by which tumour challenge within the glass cylinder leads to enhancement is not clear. It has been tentatively related to: (1) a 6-day delay in seeding of the tumour cells inoculated in a liquid environment so that the host is somehow "pretreated" with soluble tumour antigen (Filippa and Pasqualini, 1975); and/or (2) marked macrophage adherence on both the inner and outer surfaces of the glass cylinder, leading to an alteration in antigen processing (Pasqualini (1976)).

Cross-immunization between MS and histocompatible FMR leukaemia cells was described by Fefer, McCoy and Glynn (1967). In our laboratory, MS proved to be a good immunogen against the development of viral leukaemias, including the Gross + PLLV (Basombrío *et al.*, 1977). This cross-immunization was confirmed herein with a high survival rate (71%) in the MS regressors after s.c. transplantation. However, animals bearing a glass cylinder into which MS had been implanted and which had rejected it, proved to be much less immunized (22%). It can be postulated, therefore, that the glass cylinder, as a privileged site conditioning tumour enhancement, leads to a decrease in immunological memory for rejection.

### REFERENCES

BASOMBRÍO, M. A., MAYER, ALEJANDRO M. S., & PASQUALINI, C. D. (1977) Murine Sarcoma Virus Pseudotypes used as Immunogens in Viral and Chemical Oncogenesis. *Cancer Res.*, **37**, 1768.

COLMERAUER, M. E. M. & PASQUALINI, C. D. (1975) Immunological Enhancement of a Murine Allogeneic Tumour in Absence of the Spleen. *Cell. Immunol.*, **20**, 327.

CORREA, J. E., TKACZEVSKI, L. Z., COLMERAUER, M. E. M. & PASQUALINI, C. D. (1976) Murine Leukemogenic Effect of Smaller than Conventional C-type Particles. *Medicina, B. Aires*, **36**, 39.

CURRIE, G. (1976) Immunological Aspects of Host Resistance to the Development and Growth of Cancer. *Biochim. biophys. Acta*, **458**, 135.

Fefer, A., McCoy, J. L. & Glynn, J. P. (1967) Antigenicity of a Virus-induced Murine Sarcoma (Moloney). *Cancer Res.*, **27**, 962.

Filippa, D. A. & Pasqualini, C. D. (1975) Morphological Study of Allogeneic Tumour Growth in Mice Bearing a Glass Cylinder. *Medicina, B. Aires*, **35**, 29.

Kaliss, N. (1969) Immunological Enhancement. *Int. Rev. exp. Pathol.*, **8**, 241.

Kaliss, N. & Bryant, D. F. (1958) Factors Determining Homograft Destruction and Immunological Enhancement in Mice Receiving Successive Tumour Inocula. *J. natn. Cancer Inst.*, **20**, 691.

Pasqualini, C. D. (1976) Why Does a Tumour Grow? An Experimental Model for the Study of the Immunological Mechanisms Involved in Tumour Growth. *Allergol. Immunopath.*, **4**, 499.

Pasqualini, C. D. & Colmerauer, M. E. M. (1976*a*) Immunological Enhancement of a Murine Allogeneic Lymphoma. *Medicina, B. Aires*, **36**, 189.

Pasqualini, C. D. & Colmerauer, M. E. M. (1976*b*) Active Immunological Enhancement of Sarcoma 180 in Splenectomized Swiss Mice. *Biomedicine*, **25**, 202.

Pasqualini, C. D. & Colmerauer, M. E. M. (1976*c*) Tumor Enhancement in Absence of Spleen. In *Immuno-aspects of the Spleen*. Eds. J. R. Battisto and J. W. Streilein. Amsterdam: Elsevier/North-Holland Biomedical Press. p. 415.

Precerutti, A. & Law, L. W. (1963) Isolation of a Murine Leukemogenic Virus PLLV. *Nature, Lond.*, **198**, 801.

Saal, F., Colmerauer, M. E. M., Braylan, R. C. & Pasqualini, C. D. (1972) Tumour Growth in Allogeneic Mice Bearing a Plastic Cylinder. *J. natn. Cancer Inst.*, **49**, 451.

Saal, F., Colmerauer, M. E. M., Rumi, L. & Pasqualini, C. D. (1973) Conditioning Factors in Tumour Growth. *Medicina, B. Aires*, **33**, 545.